# Effect of changes to the formal curriculum on medical students’ motivation towards learning: a prospective cohort study

**DOI:** 10.1590/1516-3180.2018.0264090119

**Published:** 2019-06-10

**Authors:** Cristina Marta Del-Ben, Rosana Shuhama, Manuel João Costa, Luiz Ernesto de Almeida Troncon

**Affiliations:** I MD, PhD. Physician and Associate Professor, Department of Neurosciences and Behavior, Faculdade de Medicina de Ribeirão Preto (FMRP), Universidade de São Paulo (USP), Ribeirão Preto (SP), Brazil.; II PhD. Psychologist and Technical Health-Assistance Agent, Department of Neurosciences and Behavior, Faculdade de Medicina de Ribeirão Preto (FMRP), Universidade de São Paulo (USP), Ribeirão Preto (SP), Brazil.; III PhD. Medical Educator and Associate Professor, Medical Education Unit, School of Health Sciences, Universidade do Minho, Campus de Gualtar, Braga, Portugal.; IV MD, PhD. Physician and Full Professor, Department of Internal Medicine, Faculdade de Medicina de Ribeirão Preto (FMRP), Universidade de São Paulo (USP), Ribeirão Preto (SP), Brazil.

**Keywords:** Motivation, Learning, Curriculum, Students, medical, Education, medical

## Abstract

**BACKGROUND:**

One of the factors known to influence performance in the learning process is student motivation. In turn, students’ motivation can be regulated by a large number of variables relating to the individual (such as sex, age and socioeconomic status) or to aspects of the academic life.

**OBJECTIVE:**

The primary aim of this study was to evaluate the influence of curriculum changes involving reduction in content overload and increased early exposure to clinical settings, on motivation towards learning among Year 1 medical students. Secondarily, the aim was to ascertain whether this influence on motivation remained stable until the undergraduate program ended (Year 6).

**DESIGN AND SETTING:**

Prospective study on two student cohorts at a Brazilian state-owned university.

**METHODS:**

Two consecutive student cohorts were assessed: one with a traditional curriculum (n = 87) and the other with a reformed curriculum (n = 63), at the same medical school. Participants in both cohorts gave responses on four scales in Years 1 and 6: the Academic Motivation Scale, containing subscales for autonomous and controlled motivation, and lack of motivation towards learning; Beck’s Anxiety and Depression Inventories; Spielberger’s State-Trait Anxiety Inventory; and the Social Adjustment Scale. In Year 6, 68% of the initial sample (66 students with the traditional curriculum and 36 with the reformed curriculum) was reassessed.

**RESULTS:**

No differences between Year 1 cohorts were found regarding demographic and social background, social adjustment, depression or anxiety. Students with the reformed curriculum scored significantly higher regarding autonomous and controlled motivation than those with the traditional curriculum. Comparison between Year 6 and Year 1 showed increases in controlled motivation only for the traditional curriculum cohort.

**CONCLUSION:**

Curriculum changes were associated with increased motivation towards learning in Year 1, which persisted until Year 6.

## INTRODUCTION

Learning is influenced by different factors, and learners’ engagement is primordial among these factors. In turn, engagement is affected by students’ motivation and their perception of the relevance of the aims and features of the learning process.^1^ Consequently, well-motivated students seem to have more favorable study behavior and higher quality of learning.^1^^,^^2^

According to the self-determination proposition,^2^ motivation can be understood as a propensity to move or to do something. Motivation comprises a continuum that goes from absence of motivation at one end to extrinsic motivation and onwards to intrinsic motivation at the other end. In educational activities, extrinsic motivation is oriented towards the outcomes of the learning process, whereas intrinsic motivation is based on inherent interest in or pleasure with the process itself. Intrinsic motivation is associated with higher learning quality, increased persistence and better psychosocial adjustment.^2^

Motivation towards learning is regulated by and can itself regulate several aspects of academic life. Concerning medical education, motivation seems to influence study behavior, choice of specialty, intention to continue studying and academic success.^3^ Motivation can also be influenced by factors at the individual level that are independent of the academic environment, such as sex, age, ethnicity, socioeconomic status and personality traits.^3^

Although the influence of affective aspects of the learning process has been recognized, students’ motivation has not been a predominant driver in medical curricular planning or reform.^4^ Moreover, the effects of curricular changes on students’ motivation are not entirely understood. We previously reported^5^ that as students went through their first year in a traditional medical school, they showed significantly decreasing intrinsic motivation. These decreases were not correlated with academic performance, as measured mainly through grades in final exams. On the other hand, these students also showed higher anxiety and maladjustment of their leisure and social life at the end of the year, compared with its beginning. Potentially, these results may have been influenced by features of the formal curriculum, which was predominantly teacher-centered, with lack of awareness regarding content overload and lack of concern about clarifying the medical relevance of the scientific concepts taught, thus possibly frustrating students’ expectations.

In the light of our earlier report,^5^ the curriculum described then was reformed, guided by the SPICES model.^6^ The reform reduced the content and introduced learning activities and medical scenarios, many of them within the community, during Years 1 and 2. These activities had higher authenticity and took into consideration medical practices in the national healthcare services. The existence of two consecutive cohorts of medical students, one following a traditional curriculum and the other, a reformed curriculum, provided an opportunity to assess their possible impacts on motivation towards learning.

Therefore, the aim of this study was to evaluate whether the curriculum reform was associated with any changes in the motivation towards learning among first-year students. We also tested whether the students’ levels of motivation towards learning that had been observed in Year 1 remained stable until the end of the undergraduate program.

## METHODS

### Setting and ethics

This study was carried out at the Ribeirao Preto Medical School, a state-owned school affiliated to the University of São Paulo, in southeastern Brazil, which has an intake of 100 new students every year. The local undergraduate program committee and ethics committee approved the study (protocol 1753/2007). Some of the procedures involved here were described previously.^5^ The participants signed informed consent forms.

### Educational context

The traditional curriculum at this institution comprised didactic teaching of basic biomedical sciences (Years 1 and 2) and clinical sciences (Years 3 and 4), followed by internship training in clinical placements (Years 5 and 6). This curricular design was strongly influenced by the Flexnerian model.^7^ During the first two years, disciplines such as anatomy, physiology, genetics and biochemistry were taught using teaching and learning strategies that were based mainly on lectures and traditional laboratory activities.

The curriculum reform was restricted to Years 1 to 3, and it focused on content reduction, fostering of interdisciplinary integration and promotion of early contact with clinical activities, with increased use of the primary and secondary levels of the public national healthcare system as learning scenarios. However, the reform did not envisage any changes among academic staff members; instead, the teachers were encouraged to use active learning methods and to replace formal lectures with small-group discussions around medically relevant topics. Time was made available in the curriculum in Year 3 for teacher-student discussions of basic biomedical topics within the context of clinical activities with real patients, thus lessening the border between the basic and clinical cycles. This reform also had the aims of stimulating early engagement in discussions on issues relating to health promotion and providing a broader humanistic perspective for healthcare. Lastly, a full morning or afternoon was reserved for leisure or extra-curricular activities, in order to reduce the overload of the weekly timetable.

### Participants

Initial data were collected at the end of the academic years 1 and 6 of the two cohorts. All Year 1 medical students enrolled in the last edition of the traditional curriculum and in the first edition of the reformed curriculum were invited to participate as volunteers and asked to give responses to the instruments in a single session. Five years later, at the end of the undergraduate program (Year 6), all students who had previously taken part in the study were once again invited to respond to the self-report instruments, upon agreement through a new consent form. To preserve confidentiality, an assistant researcher who was not directly involved in the study anonymized the surveys.

### Instruments

The sociodemographic profile of the sample was assessed through a questionnaire that had been developed specifically for our previous study.^5^

The students’ social adjustment to and satisfaction with academic life, leisure activities, family relationships and financial situation were evaluated through the Social Adjustment Scale Self-Report (SAS-SR).^8^^,^^9^^,^^10^ This consists of 54 items that are rated from 0 to 5, with higher scores attributed to lower adjustment. Symptoms of depression and anxiety were evaluated using Beck’s Depression Inventory (BDI)^11^^,^^12^ and Anxiety Inventory (BAI).^13^^,^^14^ These comprise 21 items each, with scores ranging from 0 to 3. These three scales were used in their Brazilian versions, which have been shown to have good performance in reliability and validity analyses.^10^^,^^12^

In addition, in order to estimate anxiety on a trait scale, the subjects gave responses to Spielberger’s State-Trait Anxiety Inventory (STAI-T),^15^ in its version validated for use in Portuguese.^16^ The scores were classified as high if they were more than one standard deviation (SD) above the mean or low if they were more than one SD below the mean.^17^

Motivation towards learning was assessed on the Academic Motivation Scale (AMS),^18^ which has been translated into Portuguese and has been shown to have good psychometric properties.^18^^,^^19^ Each of its 28 items can be given a score between 1 (no match) and 7 (full match). These items were originally divided into seven subscales:

1. amotivation, corresponding to absence of recognition of the connection between one’s own actions and the outcome;
2. extrinsic motivation by external regulation, when the learning behavior is guided through environmental reinforcements or restrictions;
3. extrinsic motivation by introjection, which occurs through internalization of external rules;
4. extrinsic motivation by identification, when the external reasons for learning are perceived as coming from one’s own choices;
5. intrinsic motivation to experience things, guided by satisfaction that is provided through the stimulating sensations experienced during the learning processes;
6. intrinsic motivation to accomplish things, which comes from the satisfaction of acquiring new competencies to do things; and
7. intrinsic motivation to know things, corresponding to the pleasure or satisfaction in acquiring new knowledge.^18^^,^^20^

We also summarized this scale into three dimensions,^19^^,^^21^ which were named:

1. amotivation;
2. autonomous motivation (the intrinsic subscales plus extrinsic motivation by identification); and
3. controlled motivation (the two additional extrinsic motivation subscales). The coefficient of reliability (Cronbach’s alpha) was 0.83 for autonomous motivation and 0.86 for controlled motivation.^19^^,^^21^

### Statistical analysis

The statistical analysis was performed using the IBM Statistical Package for the Social Sciences (SPSS) for Windows, version 20.0 (Armonk, NY, 2011). Categorical variables were analyzed using Pearson’s chi-square test (χ^2^), and using Fisher’s exact test when appropriate. Ordinal variables were evaluated using Student’s t test. The assumed normality of the data was confirmed through the Shapiro-Wilk test.

With the aim of ascertaining changes over the time, the motivation scores were subjected to repeated-measurement multivariate analysis of variance (MANOVA) (Hotteling’s trace), considering the curricula (traditional and reformed) as “between factor” and time (Years 1 and 6) as “within factor”. Post-hoc analysis comparisons between curricula were conducted using an independent t test, and the paired Student’s t test in cases of comparison between years.

We estimated the effect size using Cohen’s d test and considered values ≤ 0.20 to be small; values > 0.20 to 0.80 to be medium, and values higher than 0.80 to be large.^22^

P-values P < 0.05 were considered significant.

## RESULTS

### Demographics, anxiety and depression

The sample was composed of 150 students: 87 (58.0%) enrolled in the traditional curriculum, and 63 (42.0%) in the reformed curriculum. The majority was male (61.3%), aged between 17 and 29 years (mean = 20.0; standard deviation, SD = 1.7).

As shown in Table 1, no significant differences were found between the students following the traditional and reformed curricula, regarding demographic, social, economic and family education background. The majority of the students classified their ethnicity as white (77.9%), had attended private high schools (89.3%) and had experienced one year or more of preparatory course for the university admission exam (71.1%). The majority of their parents (fathers 70.7%; mothers 74.0%) had attended university, and 58.8% of the students reported that their annual family income was higher than the equivalent of United States dollars (US$) 36,000.

**Table 1.** Demographic and socioeconomic background of first-year medical students enrolled in a traditional curriculum (n = 87) and in a reformed curriculum (n = 63)

	Curriculum			χ^2^	P
	Traditional n (%)	Reformed n (%)	Total n (%)		
Male	58 (66.7)	34 (54.0)	92 (61.3)	2.48	0.115
White ethnicity	67 (77.9)	49 (77.8)	116 (77.9)	1.56	0.668
Private high school	79 (89.7)	56 (88.9)	134 (89.3)	5.24	0.264
≥ 1 year of preparatory course	63 (73.3)	43 (68.3)	106 (71.1)	0.44	0.506
Father attended university	57 (65.5)	49 (77.8)	106 (70.7)	2.65	0.104
Mother attended university	61 (70.1)	50 (79.4)	111 (74.0)	1.63	0.202
Annual family income > US$ 36,000	48 (56.5)	39 (61.9)	87 (58.8)	0.44	0.507

χ^2^ = Pearson’s chi-square test; US$ = United States dollars.

In general, the participants in both curriculum groups reported having satisfactory levels of social adjustment and low levels of depressive symptoms, but had high levels of anxiety complaints (P-values ≥ 0.090; Table 2).

**Table 2.** Comparison of social adjustment, psychiatric symptoms and types of motivation between Year 1 medical students enrolled in a traditional curriculum (n = 87) and in a reformed curriculum (n = 63)

	Curriculum		P	d
	Traditional Mean (SD)	Reformed Mean (SD)		
Social adjustment (SAS-SR)				
Academic life	1.92 (0.54)	2.07 (0.56)	0.090	0.27
Leisure	1.83 (0.42)	1.99 (0.82)	0.122	0.25
Family life	1.49 (0.80)	1.71 (0.65)	0.084	0.30
Finances	1.34 (0.61)	1.36 (0.66)	0.824	0.03
Symptoms				
Anxiety (BAI)	29.6 (8.0)	28.3 (8.9)	0.485	0.21
Depression (BDI)	6.5 (6.0)	6.2 (5.1)	0.932	0.05
Intrinsic motivation (AMS)				
to know	19.7 (5.4)	22.2 (5.0)	0.005	0.48
to accomplish	15.3 (5.3)	18.8 (5.0)	< 0.001	0.67
to experience	15.4 (5.4)	17.9 (4.9)	0.003	0.50
Extrinsic motivation (AMS)				
by identification	21.7 (4.9)	23.5 (3.6)	0.017	0.41
by introjection	11.1 (5.3)	13.9 (5.7)	0.002	0.51
by external regulation	18.9 (5.9)	20.3 (5.6)	0.164	0.23
Motivation (AMS)				
Amotivation	6.6 (3.6)	7.1 (4.3)	0.447	0.13
Autonomous	72.1 (17.4)	82.4 (15.2)	< 0.001	0.63
Controlled	30.0 (9.3)	34.2 (9.7)	0.009	0.44

SD = standard deviation of the mean; SAS-SR = Social Adjustment Scale Self-Report; BAI = Beck’s Anxiety Inventory; BDI = Beck’s Depression Inventory; AMS = Academic Motivation Scale; P = Student’s t test; d = Cohen’s d test for effect size.

Through converting the BDI scores into severity classes, 89.7% of the traditional-curriculum students and 80.0% of the reformed-curriculum students were classified as having minimal presence of depression (Fisher’s exact test; P = 0.81).

Regarding the BAI classes, 70.1% and 71.0% of the students in these respective groups showed moderate presence of anxiety symptoms (Fisher’s exact test; P = 0.529). The remainder of the students in both groups showed severe anxiety symptoms (respectively 29.9 and 29.0%).

No difference in the distribution of anxiety traits was observed between the cohorts. Among the students following the traditional curriculum and reformed curriculum, 12.6% and 17.5%, respectively, were classified as having high levels of anxiety traits (χ^2^ = 0.70; degrees of freedom = 2; P = 0.704). The correlation between BAI and STAI-T scores was moderate (r = 0.542; P < 0.001).

We were able to reassess 68% of the initial samples when they reached Year 6: 66 students who were following the traditional curriculum (75.9%) and 36 who were following the reformed curriculum (57.1%). These students did not differ from the participants who had dropped out, regarding social characteristics (P ≥ 0.201) and the mean scores for social adjustment, anxiety and the seven motivation subscales (P ≥ 0.098). However, at the time when the students who were evaluated twice had entered the study, they presented fewer depressive complaints (BDI reassessed mean = 5.65, SD = 4.63, versus not reassessed mean = 7.57, SD = 6.90; P = 0.049, d = 0.33) and better academic adjustment (academic life reassessed mean = 1.87, SD = 0.46, versus not reassessed mean = 2.23, SD = 0.66; P < 0.001, d = 0.63).

### Motivation

Students enrolled in the reformed curriculum had higher mean scores on five of the motivation subscales (**intrinsic motivation to know, to accomplish things and to experience**; and **extrinsic motivation by identification** and **by introjection**) than did those following the traditional curriculum (Table 2). In other words, students enrolled in the reformed curriculum reported significantly higher scores in relation to both **autonomous motivation** (T = 3.77; P < 0.001; d = 0.63) and **controlled motivation** (T = 2.65; P = 0.009; d = 0.44) than did those following the traditional curriculum. There was no significant difference regarding the **amotivation** index.

Repeated-measurement MANOVA confirmed the differences according to curriculum that were observed during Year 1 [time and curriculum interaction, F (1,100) = 7.02; P = 0.009]. In general, students enrolled in the reformed curriculum reported higher levels of motivation, both in Year 1 and in Year 6, in comparison with students following the traditional curriculum. However, the groups of students behaved differently in Year 6 of the medical school [F (1,100) = 7.57; P = 0.007]. MANOVA applied separately to each group showed that changes to the motivation indexes occurred among the students enrolled in the traditional curriculum [F(1,65) = 7.32; P = 0.009], but not among those enrolled in the reformed curriculum [F(1,35) = 2.53; P = 0.121]. Post-hoc analyses showed that there was an increase in the **controlled** motivation of the students enrolled in the traditional curriculum (T = 2.83, P = 0.006, d = 0.39, Table 3).

**Table 3.** Results regarding motivation towards learning among medical students, expressed as mean (with standard deviation, SD), with two assessments: at the end of the first year and at the end of the sixth year of the medical school

Motivation	Traditional curriculum (n = 66)				Reformed curriculum (n = 36)			
	Year 1 Mean (SD)	Year 6 Mean (SD)	P	d	Year 1 Mean (SD)	Year 6 Mean (SD)	P	d
Amotivation	6.3 (3.0)	6.8 (3.6)	0.276	0.15	7.4 (4.8)	6.5 (3.1)	0.274	0.22
Controlled	30.3 (8.4)	33.8 (9.2)	0.006	0.39	34.5 (9.2)	33.3 (7.9)	0.362	0.14
Autonomous	73.3 (16.7)	77.7 (14.2)	0.060	0.28	83.1 (16.8)	80.9 (14.9)	0.344	0.14

SD = standard deviation of the mean; P = paired Student’s t test; d = Cohen’s d coefficient.

## DISCUSSION

The aim of this study was to assess whether changes in the early years of the formal undergraduate medical curriculum that were intended to reduce content overload and to increase early exposure to clinical and community healthcare activities, might have an impact on students’ motivation. We found that the curriculum changes were associated with increased **autonomous** and **controlled** motivations towards learning at the end of the first year, without affecting other subjective measurements, such as social adaptation, anxiety and depressive symptoms. The results further showed that the motivation levels associated with the reform in the early years were preserved throughout the program, from Year 1 to Year 6, among the students enrolled in the reformed curriculum. In contrast, we found that there was an increase in the level of **controlled** motivation measured in Year 6 among the students enrolled in the traditional curriculum, such that it became similar to what had already been seen in Year 1 among the students enrolled in the reformed curriculum.

The demographic profiles of the medical students included in our study were similar to what had previously been reported in our medical school^23^ and in other medical schools in Brazil.^19^^,^^24^ In Year 1, both groups showed satisfactory levels of social adjustment and low levels of depressive symptoms, but showed levels of anxiety complaints that ranged from moderate to high, along with anxiety traits. These levels were observed independently of the curriculum that was followed. It is likely that this finding can be explained by the fact that the measurements were made at the end of the academic year, when students are usually concerned about the proximity of the final exams.

The pattern of motivation towards learning among our medical students was also similar to what was obtained using the same instrument in another Brazilian medical school, 10 years earlier.^19^ The highest score was obtained on the subscale **extrinsic motivation by identification** followed by the score for **intrinsic motivation to know**; whereas **amotivation** showed the lowest score, followed by the score for **extrinsic motivation by introjection**. However, the mean scores obtained from our sample were lower than those described previously,^19^ particularly among the students enrolled in the traditional curriculum.

The higher levels of motivation observed among the students enrolled in the reformed curriculum can be correlated with some of its new features, which fitted into Harden’s SPICES model.^6^ In the new curriculum, greater student-centeredness was achieved through reductions in content overload and the encouragement that was given for teachers to use small-group discussions instead of formal lectures. Greater integration between fields was pursued through merging traditional disciplines into broader modules, such as “general morphology”, covering anatomy, histology and embryology. Early clinical experience for students was planned to occur predominantly within community healthcare settings.

Because the reform touched several dimensions of the SPICES model, it is not possible to identify key elements that would be particularly associated with increased student motivation. All of the improvements were likely to have contributed towards increasing the motivation towards learning. For instance, a review of earlier literature on the psychological basis of problem-based learning (PBL), an educational strategy that encompasses many of the SPICES model components, found indirect evidence of increased “intrinsic interest in the subject matter”, which is clearly a construct relating to motivation.^25^ Another review on PBL outcomes found evidence of increased student satisfaction with their learning processes and environment.^26^ Along the same lines, another study showed that students enrolled in a PBL curriculum were more likely to have intrinsic motivation, whereas those following a traditional curriculum tended to express extrinsic motivation, particularly in the first years of the medical school.^27^ Furthermore, a systematic review showed that there were associations between early experience for students in clinical and community settings, which was a major feature of the reform reported here, and a number of positive outcomes, including increased student motivation, through “**reminding them of their vocation to be a doctor and reinforcing it**” and therefore enhancing learning using a variety of mechanisms.^28^

One perspective that is complementary to this is that newly admitted Year 1 medical students may have perceived the curriculum changes as evidence of institutional commitment to their education. This is one of the most important factors determining students’ success, according to Tinto and Pusser’s model.^29^ This model was developed to reach better understanding of the factors involved in students’ attrition, persistence and success, but the motivational component was implicit in many of its considerations. Moreover, students’ perceptions relating to the quality of the course, in terms of the meaning and value of the educational experience, have also been regarded as linked to autonomous motivation.^2^

Among the students who followed the traditional curriculum, **controlled** motivation increased significantly from Year 1 to Year 6. This can be explained by the fact that learning activities during Years 3-6 were almost exclusively carried out within healthcare settings, thus fulfilling student expectations. On the contrary, there were no significant changes over the course of time on any of the motivation subscales, among the students following the reformed curriculum. Since the changes associated with the curricular reform predominantly affected the first two years of the undergraduate medical program, with only minor changes in Year 3 and no impact on Years 4-6, it is unlikely that the students’ motivation towards learning would have continued to increase. Nevertheless, these findings, taken overall, indicate that the learning activities in each of the years (Year 2 to Year 6) were able to sustain the students’ increased level of motivation towards learning that were associated with the curricular reform.

Some caveats should be considered before generalizing the findings from this study. Our results need to be analyzed cautiously taking into account the small size of the samples and the use of a single school as the recruitment context for the volunteers who were studied. Particularly regarding the follow-up, there may also have been some bias towards presumably better adapted individuals, since the students who we reassessed in Year 6 were the same who had better academic adjustment and lower scores for depressive complaints at the end of Year 1. Indeed, regarding this last point, despite the lower scores, no volunteer was classified as having more than a minimal level of presence of depressive symptoms. Furthermore, other than a trend relating to anxiety, which seems to influence motivation,^30^ personality traits and other markers of psychological stress and indicators of academic performance were not assessed. Thus, the implications of these factors for changes in motivation could not be determined. Nevertheless, our study provides a contribution given that, so far, few studies have studied the impact of curricular changes on motivation towards learning among medical students, using validated instruments and a prospective design.

In summary, our results indicate that a curriculum reform that was designed mainly to reduce content overload and provide early medical experiences in community settings was associated with increased motivation towards learning among freshman medical students. Our data also suggest that changes to the curriculum in the early years of the undergraduate program may bring forward the higher motivation levels that would otherwise only be reached later. These findings should be considered when making decisions on the changes to be included in curriculum reform within more traditional undergraduate medical programs.
